# Limited trophic partitioning among sympatric delphinids off a tropical oceanic atoll

**DOI:** 10.1371/journal.pone.0181526

**Published:** 2017-08-02

**Authors:** Hillary Young, Katherine Nigro, Douglas J. McCauley, Lisa T. Ballance, Erin M. Oleson, Simone Baumann-Pickering

**Affiliations:** 1 Department of Ecology, Evolution and Marine Biology, University of California Santa Barbara, Santa Barbara, CA, United States of America; 2 Southwest Fisheries Science Center, NOAA Fisheries, La Jolla, CA, United States of America; 3 Scripps Institution of Oceanography, University of California San Diego, La Jolla, CA, United States of America; 4 Pacific Islands Fisheries Science Center, NOAA Fisheries, Honolulu, Hawaii, United States of America; Stockholm University, SWEDEN

## Abstract

Understanding trophic relationships among marine predators in remote environments is challenging, but it is critical to understand community structure and dynamics. In this study, we used stable isotope analysis of skin biopsies to compare the isotopic, and thus, trophic niches of three sympatric delphinids in the waters surrounding Palmyra Atoll, in the Central Tropical Pacific: the melon-headed whale (*Peponocephala electra*), Gray’s spinner dolphin (*Stenella longirostris longirostris*), and the common bottlenose dolphin (*Tursiops truncatus*). δ^15^N values suggested that *T*. *truncatus* occupied a significantly higher trophic position than the other two species. δ^13^C values did not significantly differ between the three delphinds, potentially indicating no spatial partitioning in depth or distance from shore in foraging among species. The dietary niche area—determined by isotopic variance among individuals—of *T*. *truncatus* was also over 30% smaller than those of the other species taken at the same place, indicating higher population specialization or lower interindividual variation. For *P*. *electra* only, there was some support for intraspecific variation in foraging ecology across years, highlighting the need for temporal information in studying dietary niche. Cumulatively, isotopic evidence revealed surprisingly little evidence for trophic niche partitioning in the delphinid community of Palmyra Atoll compared to other studies. However, resource partitioning may happen via other behavioral mechanisms, or prey abundance or availability may be adequate to allow these three species to coexist without any such partitioning. It is also possible that isotopic signatures are inadequate to detect trophic partitioning in this environment, possibly because isotopes of prey are highly variable or insufficiently resolved to allow for differentiation.

## Introduction

Relative to higher latitude ecosystems, tropical ocean environments are generally resource poor, and prey patches thinly distributed and often unpredictable in space and time. Within these ecosystems, oceanic islands can provide centers of increased productivity. Termed the “island mass” effect [1–3], this productivity can result from island wakes, eddies, fronts, and other physical features that enhance primary productivity, passively concentrate planktonic prey, and attract upper trophic-level predators. Odontocetes (toothed whales) are among marine predators known to be both abundant and diverse near islands relative to surrounding oceanic waters [4–8], raising questions pertaining to the mechanisms in general that allow for their coexistence in higher abundance and diversity than surrounding open ocean waters. Resource partitioning is an important way in which competition is minimized among species, and has been documented among seabirds and fish predators in these remote and low productivity systems [9–12]. However, a number of studies also document a high degree of trophic overlap in oceanic oligotrophic waters for a number of marine predators including sharks and seabirds [13–15].

The amount of resource partitioning within and between coexisting species groups is reflected in the size and diversity of each group’s niche, which includes habitat, food-type, and temporal dimensions [16]. The extent of interspecific and intraspecific competition for resources in a community alters these dimensions for each species, resulting in changes in their niche characteristics [16, 17]. Heightened interspecific competition, due to an increase in consumers or a lack of resources, is thought to reduce the size and variability of a population’s niche, as individuals within that population are forced to feed from a reduced selection of resources [17, 18]. Conversely, when interspecific competition is reduced and/or resources are abundant, population niches are expected to expand–either by all individuals within the population consuming a larger diversity of resources or by individuals specializing on different resources, cumulatively expanding the population’s trophic niche [17, 19].

In both high- and low-productivity marine ecosystems, coexisting odontocete populations have been documented to exhibit varying degrees of resource partitioning. In higher productivity systems, where resources should be abundant, evidence for interspecific habitat and prey partitioning has been discovered [20–22]. Similarly, intraspecific resource and habitat partitioning has also been documented in odontocetes based on age, size and sex of individuals [23, 24]. There has been some evidence of resource overlap between odontocetes in a high productivity system; this is possibly because resources were so abundant as to not be limiting in this system [25].

In less productive waters, the degree to which odontocete niche partitioning occurs is less clear. In the eastern tropical Pacific, spotted dolphins (*Stenella attenuata*) and spinner dolphins (*Stenella longirostris*) appear to partition resources interspecifically by foraging at different times, at different depths, and on different prey [26, 27]. Similarly, niche partitioning by depth was found within an odontocete community in the Bahamas [28]. The two most extensive studies in tropical oceanic islands both found evidence of niche partitioning within the delphinid community—either by habitat, feeding times, or feeding depths [7, 29].

A useful tool for evaluating resource partitioning within and among species is carbon and nitrogen stable isotope analysis (SIA), which has the advantage of integrating diet information over multiple months as well as providing information on the type and location of resources utilized [30]. By quantifying the stable nitrogen and carbon isotopic ratios (^15^N/^14^N, annotated δ^15^N and ^13^C/^12^C, annotated δ^13^C) in animal tissue, the trophic position of that animal can be approximated as heavier isotopes are retained while lighter ones are secreted [31]. Therefore, nitrogen and carbon stable isotopes of consumers reflect those of their prey. A biplot of consumers’ isotopic signatures, representing each population’s isotopic niche, can then provide information related to the trophic niche width, individual variability in resource use, and amount of overlap in niches between populations [18, 22, 30, 32, 33].

Fractionation of nitrogen across animal species varies widely by taxonomic group, but has relatively small variation within mammals, which have an average increase of ~+2.5‰ per trophic exchange [34]. However, recent work on *T*. *truncatus* suggested that the value may be much lower for this species (1.6 ‰) and possibly for other delphinids as well [35, 36]. In this study, it was assumed that variation in δ^15^N is due primarily to isotopic fractionation, providing an indication of variation in trophic position. Variation in δ^15^N from terrestrial runoff from anthropogenic sources, are not relevant to this system as there is no nearby human settlement [37].

Carbon stable isotope values are mainly determined by the manner in which carbon was originally fixed [38]. In marine ecosystems, carbon stable isotope values are generally enriched in nearshore, benthic environments and depleted in offshore pelagic environments [39]. This is ultimately driven by differences in the photosynthetic rates of primary producers in benthic as compared to pelagic environments [39]. In nutrient-rich benthic environments, primary producers grow more rapidly and take up more ^12^C during photosynthesis, resulting in an increase in their δ^13^C values. In pelagic environments, lower nutrient levels lead to lower growth rates and lower δ^13^C values. In contrast to this spatially-driven δ^13^C gradient, trophic fractionation of carbon isotopes is likely small. Thus, δ^13^C in this study is used as an indicator of foraging habitat (distance from coast and depth of foraging) rather than trophic position.

SIA is a useful tool for understanding foraging ecology of odontocetes because skin biopsy samples can be obtained from free-ranging animals using methods that are relatively non-invasive with negligible impact; isotopic results from these skin biopsies are comparable to those from other types of samples used for SIA, such as teeth and muscle samples [40, 41]. For epidermal tissue, SIA reflects the diet of the animal during the period of skin growth. While the turnover time for isotopes in cetacean skin tissues has not been extensively studied, isotopic turnover in *T*. *truncatus* estimates a half-life of 24 (carbon) and 48 (nitrogen) days, suggesting that the skin will integrate feeding data over a period of several months [35]. Full turnover (>95%) appears to take about 104 days for carbon and 205 days for nitrogen [35], providing a high-end estimate of isotopic persistence. While this is likely to be similar for other delphinid species, it is possible that there could be some differences in skin turnover or seasonal variation in skin growth among delphinid species. However, it is likely to integrate over long time periods (multiple months) for all species considered.

Here, SIA was used to examine the diet overlap of the three most common cetacean species found in waters around Palmyra Atoll in the Line Island chain in the central Pacific: *P*. *electra*, *S*. *longirostris*, and *T*. *truncatus*. We investigate the degree to which isotopic evidence indicates that inter- and intraspecific trophic niche partitioning occurs among these three species, and offer some additional data to clarify the significance of competition and niche partitioning in structuring odontocete communities around tropical oceanic islands.

## Materials and methods

### Study site

This work was conducted in the waters of Palmyra Atoll National Wildlife Refuge (5.867 N, 162.067 W), a very large marine protected area in the central tropical Pacific. It is part of the Line Islands chain; the nearest emergent land masses in this chain are > 60 km away. The waters surrounding Palmyra Atoll are warm (25–30°C), with low surface chlorophyll concentration (chlorophyll *a* 0.1–0.2 mg m^3^), and deep, sloping rapidly to 1,000–5,000 m within 0.5 km of the shore with limited bathymetric features in surrounding waters [10]. Nutrient runoff from terrestrial inputs results in higher productivity immediately surrounding the atoll [11]; island upwelling effects likely exaggerate this. Palmyra is located within the Inter-tropical Convergence Zone (ITCZ), a meteorological feature that is typically associated with calm winds and frequent rainfall. Interannual climate variation at Palmyra is driven largely by the El Niño-Southern Oscillation (ENSO), which causes significant sea surface temperature anomalies during El Niño years [42]; some such anomalous warming occurred in 2009.

The marine mammal community in the nearshore waters of Palmyra Atoll is dominated by three species (*Peponocephala electra*, *Tursiops truncatus*, and *Stenella longirostris*) but also regularly includes at least one beaked whale species, Deraniyagala’s beaked whale (*Mesoplodon hotaula*) [43]. Other species known to be present at least occasionally are Blainville’s beaked whale (*Mesoplodon densirostris)*, Cuvier’s beaked whale (*Ziphius cavirostris*) [44], Longman’s beaked whale (*Indopacetus pacificus*), short-finned pilot whale (*Globicephala macrorhynchus*), false killer whale (*Pseudorca crassidens*), killer whale (*Orcinus orca*), rough-toothed dolphin (*Steno bredanensis*), Bryde’s whale (*Balaenoptera edeni*), *Stenella attenuata*, *Stenella coeruleoalba*, and *Physeter macrocephalus* [45].

Surveys were conducted for cetaceans near Palmyra Atoll once or twice per year, generally in spring and fall, from 2009 to 2012. Survey tracks near the atoll were not systematic, but were intended to cover available nearshore habitat, surveying waters from 100 m to 10 km from shore and 20 m to 3 km depth where animals were often easily encountered during the day (see S1 Fig for a map of all survey tracks by year). At each encounter, the species were identified and information on location, time, observational cue, group behavior, group size, group composition, weather and sea condition, number of animals approached within 100 m and potential behavioral change due to approach were noted. Individuals were photographed using Single-lens reflex (SLR) cameras with telephoto lenses. Identification photos collected during cetacean encounters suggest that both *P*. *electra* and *T*. *truncatus* may remain longer term, at least within the month of a survey, based on repeated sightings of individuals [46]. *P*. *electra* were not observed during the fall survey in 2009, nor during opportunistic observations by other researchers at the atoll towards the end of that year, suggesting that possible residency may be intermittent.

### Sample collection

From 2008 to 2011, biopsy samples were collected each year between the months of May and November from encountered cetaceans during good sea conditions (Beaufort <3) during the day. The samples were collected using a Barnett Wildcat crossbow (110 lb. draw limb) that fired free-floating darts with 25 mm cutting heads, which extracted a plug of tissue about the size of a pencil eraser and were picked up after they bounced off the targeted animal. All tissue samples are permanently archived at Southwest Fisheries Science Center in La Jolla, California. Samples were stored in -20°C prior to laboratory analysis. For each sample, we recorded associated group sighting information, age class of biopsied individual (subadult or adult), location of biopsy on body and behavioral response to the biopsy. Photos of the individual were taken if possible.

### Sample preparation and analysis

Each biopsy sample was split, with half retained for genetic analysis and half utilized for isotopic analysis. For the sample used for isotopes, skin tissue was manually removed from the blubber, finely chopped and then freeze-dried for 72 hours. As variation in lipid levels among samples can alter δ^13^C values substantially [36], lipid was removed using a solution of dichloromethane: methanol (9:1) and sonication, followed by evaporation at 65°C; this process was replicated three times per sample to ensure removal of all lipids [47, 48]. Samples were then ground to a fine powder. Stable C and N isotopes of the 2009 and 2010 samples were analyzed using a Finnigan Delta Plus XP isotope ratio mass spectrometer coupled to a Carlo Erba NA 1500 Series 2 elemental analyzer. Replicate laboratory standards of graphite NIST RM 8541 (USGS 24) and ammonium sulfate NIST RM 8547 (IAEA N1) were used within each run. The rest of the samples (from 2008 and 2011) were run at the UC Davis Stable Isotope Lab using a PDZ Europa ANCA-GSL elemental analyzer interfaced to a PDZ Europa 20–20 isotope ratio mass spectrometer (Sercon Ltd., Cheshire, UK), with a standard deviation of 0.2 ‰ for ^13^C and 0.3 ‰ for ^15^N. The elemental composition of C and N was used to calculate sample C:N ratio, with a C:N ratio <4 considered indicative of effective lipid removal [49].

### Statistical analyses

In order to assess inter- and intraspecific resource partitioning, multivariate analysis of variance (MANOVA) was used, followed by univariate ANOVA tests to test for absolute differences in δ^13^C and δ^15^N among species. Then post-hoc Tukey honest significant difference (HSD) analyses were used to identify pairwise differences between species and years for the two most commonly sampled species (*P*. *electra* and *T*. *truncatus*). Prior to analysis, data were first tested for normality and homoscedasticity.

Trophic niche width between species was compared using the Stable Isotope Bayesian Ellipse technique [33] which accounts for biases due to small and variable sample sizes, which otherwise tend to strongly bias estimates of niche width using traditional metrics [32]. The SIBER package in R, which employs Markov-Chain Monte Carlo (MCMC) simulations, was used to construct parameters of ellipses based on sampling points. The standard ellipse area (niche breadth) for each species was estimated based on 10,000 posterior draws and corrected for small sample size [33]. The probability that two niche areas differed from each other was determined using Bayesian inference based on the 10,000 posterior draws (i.e. the probability that the niche area of group 1 is greater than group 2 is the proportion of group 1 standard ellipses that are greater than group 2 standard ellipses, based on the 10,000 replicates). Plotted ellipses are maximum likelihood standard ellipses corrected for small sample sizes; traditional standard convex hulls are also plotted [32]. Percent overlap in niches was calculated using the SIAR package in R (with 100% as the upper limit) and was determined using these small sample size-corrected ellipses. While the *O*. *orca* sample is shown in plots with raw data, it is not included in any analyses given that there was only a single sample. Data are shown and reported as means ± 1 SD unless otherwise indicated.

### Permitting

This work was permitted by US Fish and Wildlife Service (12533–08001) and by National Marine Fisheries Service (774–1714). This work was approved by Institutional Animcal Care and Use Committee (IACUC) of University of California San Diego, Scripps Institute of Oceanography (approval number S08223). No anesthesia, euthanasia, or any kind of animal sacrifice were part of the study. All sampling procedures were reviewed as part of obtaining the appropriate permits.

## Results

A total of 110 animals were sampled. Samples were collected during five sampling periods spanning four years and six months: Aug and Sept 2008 (n = 34), Sept and Oct 2009 (n = 8), June and August 2010 (n = 57), May 2011 (n = 6), Nov 2011 (n = 4), from *P*. *electra* (n = 45), *T*. *truncatus* (n = 53), and *S*. *longirostris* (n = 11). One sample that was collected from *O*. *orca* in August 2010 is also included as an anecdotal reference sample for this species. Sample size by species and year are summarized in S1 Table.

The mean C:N ratio of the 110 samples analyzed was 3.31. Using replicate standards across and within runs, analytical error was less than 0.2‰ for both C and N.

**Fig 1:**
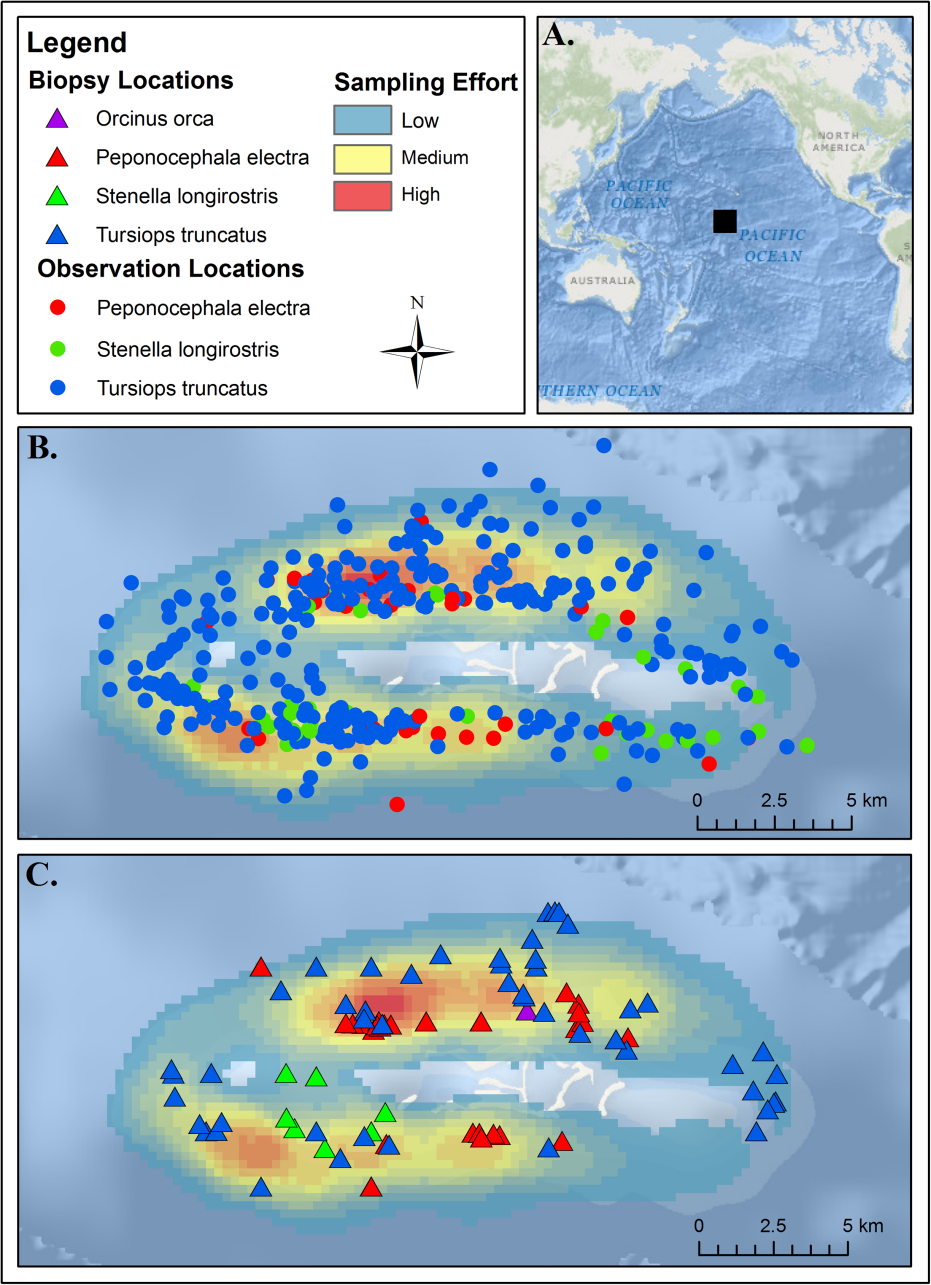
Sighting and sampling locations for each delphinid species. All sampling was conducted at Palmyra Atoll in the Northern Line Islands in the Central Pacific Ocean (A). Panel B shows all sighting locations (circles) and Panel C shows all sampling locations (triangles) of each delphinid species across all years. Full ship survey tracks are in S1 Fig; however given the heavy overlap of tracks, as an index of relative sampling density across areas, we here visualize this effort by means of a heat map of sampling effort (interpolated from raw ship tracks across all years using Point Density Spatial Analyst Tool in ArcMap 10.2.1). Source credits for bathymetric maps are Esri, DeLorme, GEBCO, NOAA and other contributors.

On average (across all years), *Stenella longirostris* was sighted and sampled at shallower depths (mean 180 m depth for all sightings) than were either *Tursiops truncatus* (mean 584 m depth) or *Peponocephala electra* (mean 548 m depth). The distance sampled from shore was slightly further for *T*. *truncatus* (3200 m mean) as compared to either *P*. *electra* (2455 m) or *S*. *longirsotris* (2630 m) (Fig 1). However, we do not believe that this daytime sighting data can be used to understand spatial niche partitioning in feeding behavior; indeed all sampling was taken during daytime, but at least some of these species are known to engage heavily in nighttime feeding and may move offshore towards evening. A strength of stable isotope analysis is that it can help overcome the challenges otherwise associated with identifying feeding areas for animals that feed primarily at night when they are not easily observed.

### Interspecific niche differentiation

**Fig 2:**
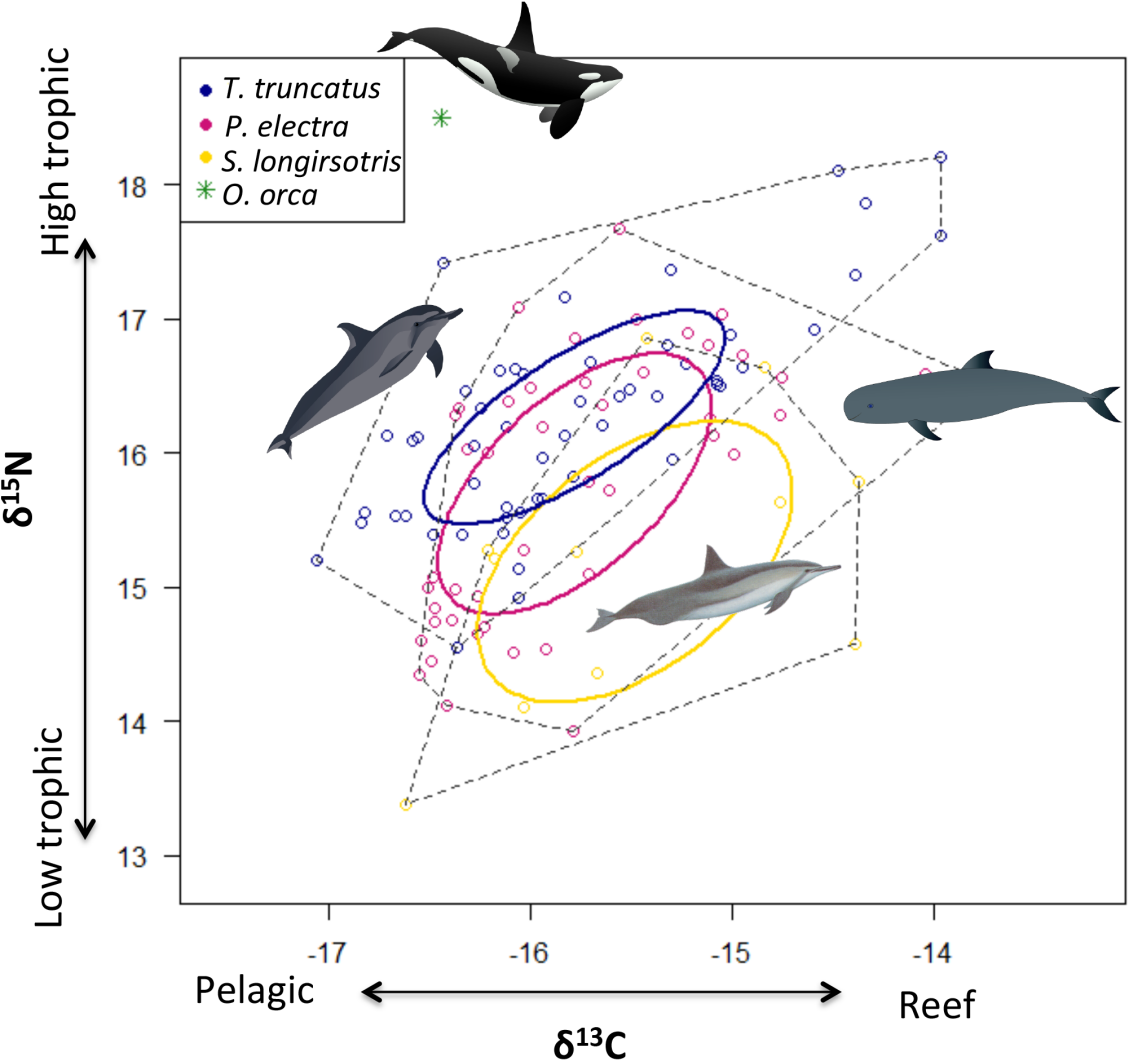
Isotope space plot for each delphinid species. δ^13^C and δ^15^N isotopic values for the three most common cetacean species, as well as the single individual of *O*. *orca*, pooled across all years (2008–2011), at Palmyra Atoll. Dashed lines show convex hulls for all samples, while thick colored lines show 95% CI bivariate ellipses (see Methods).

The three species of cetaceans showed some isotopic partitioning (MANOVA, Wilk’s lambda, F_2, 106_ = 8.99, p<0.0001; Fig 2). Univariate tests revealed that this variation was due almost entirely to differences in δ^15^N (ANOVA, F_2, 106_ = 8.09, p < 0.001; Table 1), as there were no significant differences in δ^13^C among the species (ANOVA, F_2, 106_ = 0.86, p = 0.43; Table 1). Results from post hoc Tukey pairwise comparisons show that these differences were largely due to δ^15^N of *T*. *truncatus* being significantly higher than both *P*. *electra* (p < 0.05) and *S*. *longirostris* (p < 0.01). The δ^15^N values of these latter two species did not significantly differ from each other (p = 0.14; Table 1).

**Table 1:** Mean isotopic values for each delphinid species.

Species	n	δ^15^N	δ^13^C
*T*. *truncatus*	53	16.3 ± 0.8^A^	-15.8 ± 0.8^A^
*P*. *electra*	45	15.8 ± 1.0^B^	-15.8 ± 0.7^A^
*S*. *longirostris*	11	15.2 ± 1.1^B^	-15.5 ± 0.8^A^

Mean δ^13^C and δ^15^N with standard deviation for three delphinid species at Palmyra Atoll, pooled across years (2008–2011). Superscript letters indicate significant differences between species in post hoc analyses; values not sharing a letter are significantly different.

### Interspecific dietary niche width and overlap

**Fig 3:**
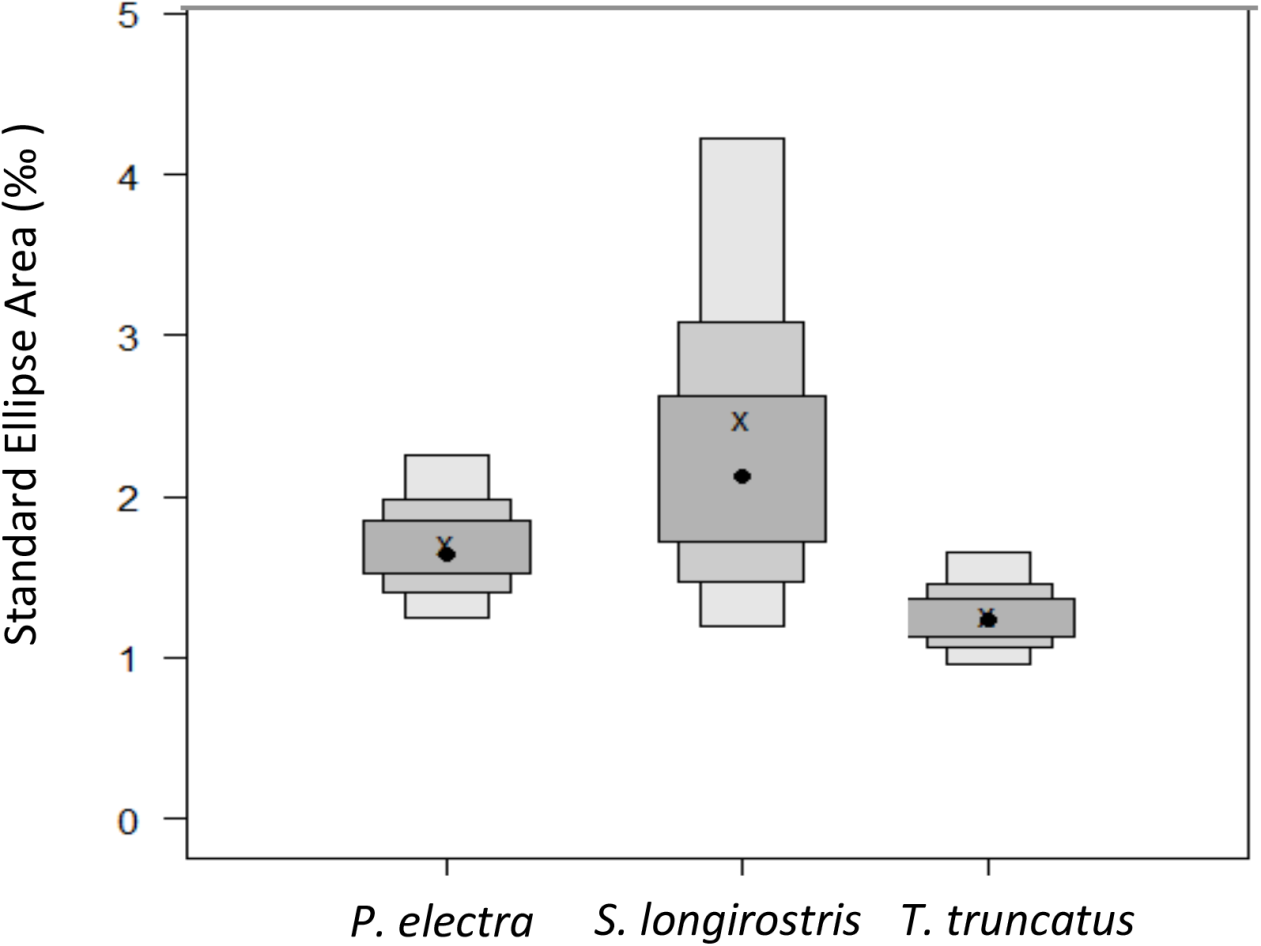
Niche width of each delphinid species. Niche width (calculated using measures of uncertainty for Bayesian standard ellipse areas) for three delphinids at Palmyra Atoll, pooled across all years (2008–2011). Black dot represents the central tendency with 95, 75, and 50% credibility intervals (shading from light to dark grey). “X” indicates the mean standard ellipse area of each species using a non-Bayesian, but sample size-corrected approach.

Dietary niche width, as measured via stable isotope analysis, varied between species, with *S*. *longirostris* having a slightly larger estimated niche width than *P*. *electra;* both species had substantially larger niche widths than *T*. *truncatus* (Fig 3). The niche area of *S*. *longirostris* was 24.9% larger than that of *P*. *electra* (SEAc = 2.5 ‰ and 1.9 ‰, respectively; probability of true difference = 0.84 based on 10,000 posterior draws- see Methods). The dietary niche area of *T*. *truncatus* (SEAc = 1.29 ‰^2^) was 30.6% smaller than that of *P*. *electra* (probability = 0.93) and 47.8% smaller than that of *S*. *longirostris* (probability = 0.98).

There was a total of 39.5% overlap between all three species (Fig 2). The highest dietary niche overlap (36.4%) occurred between *P*. *electra* and *T*. *truncatus*. Niche overlap was 24.1% between *S*. *longirostris* and *P*. *electra* and only 2.3% between *S*. *longirostris* and *T*. *truncatus*.

### Intraspecific niche differentiation

**Fig 4:**
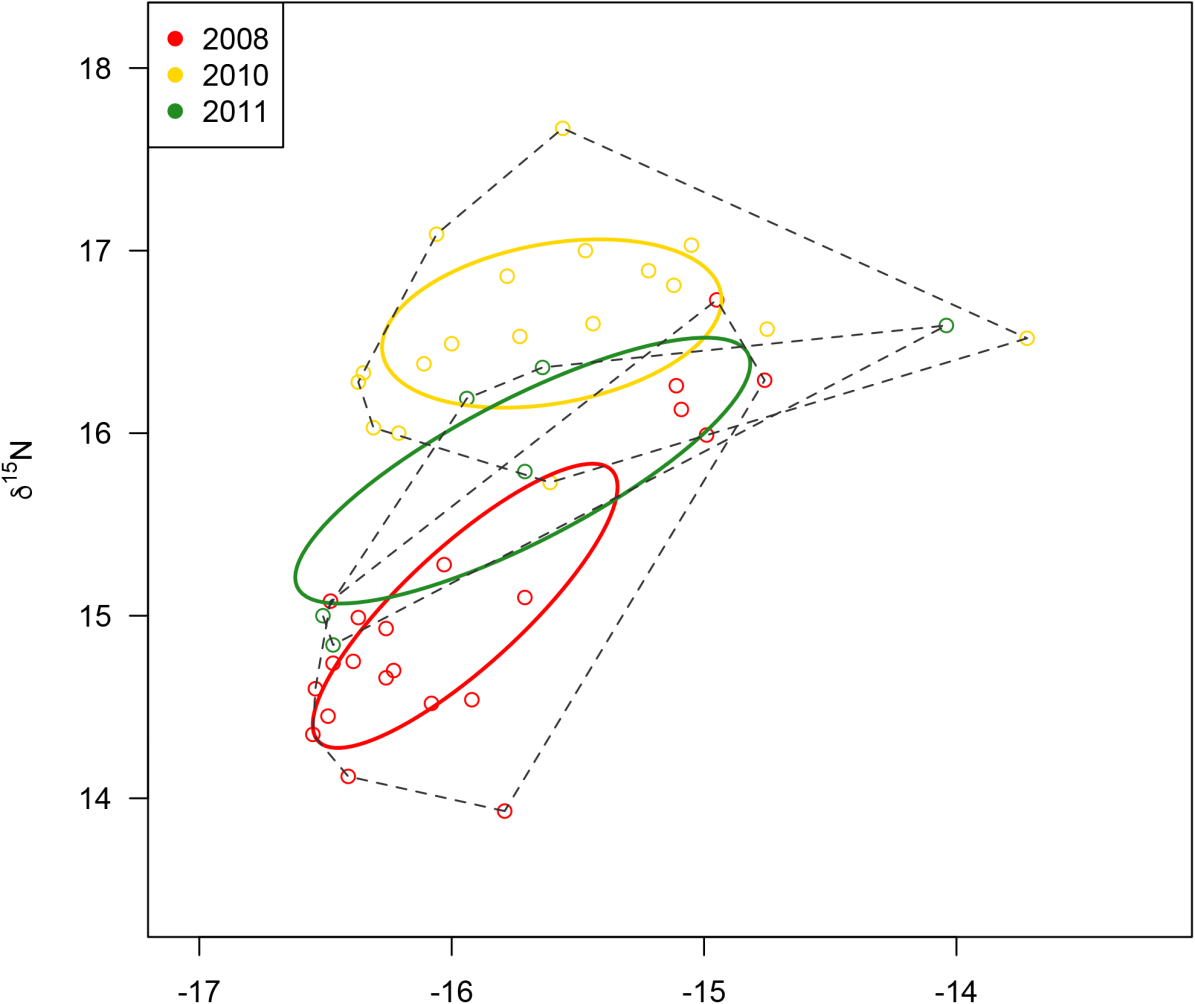
Interannual isotope space plot for *P*. *electra*. Variation in δ^13^C and δ^15^N isotopic values of *P*. *electra* by year. Dashed lines show convex hulls for all samples, while thick colored lines show 95% CI bivariate ellipses (see Methods).

*Peponocephala electra* was the only species with significant niche differentiation between the years it was sampled (2008, 2010 & 2012) (MANOVA, Wilk’s lambda, F_2,42_ = 12.85, p<0.0001; Fig 4). This was due to differences in δ^15^N (ANOVA, F = 26.47, p<0.0001) rather than δ^13^C (ANOVA, F = 1.29, p = 0.29). The mean δ^15^N value for *P*. *electra* was significantly higher in 2010 than in 2008 (p<0.01) and 2011 (p<0.05); the latter two years could not be distinguished from each other (p = 0.051). Bivariate niche space analysis indicated little overlap in *P*. *electra* niche between years (Fig 4), with 7.6% overlap between 2010 and 2011, 13.7% overlap between 2008 and 2011 and no overlap between 2008 and 2010. *P*. *electra*’s niche area was greater in 2011 than in 2008 (probability = 0.91) and in 2010 (probability = 0.88).

## Discussion

### Interspecific niche differentiation

A significantly higher δ^15^N signature for *Tursiops truncatus* was detected compared to *Peponocephala electra* and *Stenella longirostris*, potentially suggesting that *T*. *truncatus* may feed on higher trophic position prey than do the other two delphinids. However, this effect was very small; assuming an average δ^15^N enrichment of 2.5 ‰ with each trophic level [34], the difference indicates that *T*. *trucatus* is one fifth of a trophic level higher than the other delphinids. To our knowledge, no other studies have compared the trophic ecology of *T*. *truncatus* to either *P*. *electra* or *S*. *longirostris* using isotopes, although these three species are sympatric in many regions. Both *P*. *electra* and *S*. *longirostris* are known to forage at night, primarily on deep scattering layer-associated organisms that come to the surface as part of diel vertical migrations [29, 50–52]. In contrast, *T*. *truncatus* is thought to forage diurnally [53, 54] and rely more heavily on demersal and benthic fish and squid [55], making it unlikely that they forage sympatrically with the other two delphinid species. However, this cannot be entirely ruled out as *T*. *truncatus* populations in other areas, such as the Atlantic offshore ecotype, have been documented to forage heavily at night, presumably also on mesopelagic fish and squid that migrate to surface waters [56]. As *S*. *longirostris* is somewhat smaller than the other two delphinid species examined [52] it may thus target prey of a smaller size, which could explain its relatively lower δ^15^N signature compared to *T*. *truncatus*. Indeed, a correlation between trophic position and body size was found in a study of Moorea’s delphinid community [8]. However, mesopelagic fish are generally small and it is not clear that even the largest fish would be too big for *S*. *longirsotris*.

Consistent with broad scale dietary observations that show *P*. *electra* and *S*. *longirostris* feed on generally similar groups and size classes of prey [26, 50, 51], there were no differences in δ^15^N between these species. Our results are similar to those from Moorea (near Tahiti), which found no statistically significant differences in isotopic position between these two species [8].

There were no significant differences in δ^13^C among the three species examined, indicating that they feed on prey that is similarly enriched in δ^13^C, and potentially indicating similar prey sources. At least for *S*. *longirostris* and *P*. *electra*, this is highly consistent with observations elsewhere, which show *P*. *electra* and *S*. *longirostris* engaging in similar feeding behaviors, where they rest near shore during the day and move offshore to forage on vertically migrating prey at night [51, 57]. The lack of δ^13^C variation in this habitat may simply be due to lack of extensive onshore feeding opportunities. Palmyra Atoll has a steep and narrow insular slope, such that there are very limited nearshore waters in an otherwise oceanic system. There may also be limited variation in δ^13^C sources in this system. However, other studies at Palmyra Atoll among multiple groups of marine predators (seabirds, sharks and other fish) with known residency or movement behaviors have shown strong variation in δ^13^C correlated to distance of foraging off shore; making these explanations somewhat unlikely [9,10,12]. It is also possible that different dietary compositions of prey with substantial variation in δ^13^C by chance resulted in similar δ^13^C average values, thereby masking real differences. Stomach content analysis would be useful in investigating this possibility further; however, these data are currently not available and may be unlikely to obtain for a remote location such as Palmyra Atoll. Lastly, we should note that methodologically it is also possible there may be subtle species specific differences in discrimination factors (e.g., due to differences in metabolic or growth rates) that are not currently known. These could drive differences in isotopic values across species, although there is no current evidence to suggest this is the case.

### Interspecific dietary niche width

*Tursiops truncatus* had a smaller dietary niche area than both *P*. *electra* and *S*. *longirostris*, which suggests that there is less intraspecific variability in diet between individuals in the population and that there is more interspecific competition for resources or less resources available, resulting in a more specialized dietary niche than the other two species [17, 58] (Fig 3). This is surprising as throughout its large range *T*. *truncatus* is known to eat a variable diet and adapt its foraging strategy to its environment and prey, making it appear to be a generalist [52, 53, 59]. However, while *T*. *truncatus* can appear to be a generalist on a global scale, at localized scales their diets are often somewhat specialized [52]. Therefore, the population of *T*. *truncatus* near Palmyra Atoll may be composed of a group of individuals that similarly specialize on the same prey groups, while the populations of *P*. *electra* and *S*. *longirostris* contain either more individual specialization or more generalist individuals [58]. The smaller niche area of *T*. *truncatus* could also reflect its different and potentially more diurnal foraging strategy; it is possible that the mesopelagic fish and squid that are the nocturnal prey of *P*. *electra* and *S*. *longirostris* are simply a more diverse group than the prey available to *T*. *truncatus*.

### Niche overlap

The large amount of overlap between *T*. *truncatus* and *P*. *electra* suggests that, despite significant if small differences in δ^15^N, there is only limited niche partitioning occurring. It may be that the temporal niche differences among these species may minimize direct competition and the need for other forms of resource partitioning. While *T*. *truncatus* is known to be active during both day and night with feeding peaks in the early morning and late afternoon [53, 54], *P*. *electra* and *S*. *longirostris* instead rest during the day and forage at night [29, 50, 51, 52]. In contrast *T*. *truncatus* had very little niche overlap with that of *S*. *longirostris*, despite the relatively large niche width of this latter species. The lack of overlap was driven by the much lower δ^15^N values of *S*. *longirostris*, suggesting that they may partition resources by feeding on different prey populations.

Not surprisingly, given the lack of differences in both δ^15^N and δ^13^C between *S*. *longirostris* and *P*. *electra*, and the relatively large niche areas of these two species, there was also strong overlap between *S*. *longirostris* and *P*. *electra*. While this is consistent with the joint foraging in space and time reported in other studies [7, 29], it either suggests a lack of direct competition, perhaps due to an abundance of resources, or a nuanced partitioning of prey type beyond what can be detected with integrative approaches such as stable isotopes.

### Intraspecific niche variation between years

Of the two delphinid species that were well sampled at Palmyra Atoll across multiple years (*P*. *electra* and *T*. *truncatus*), one of these—*P*. *electra—*showed significant variation in isotopic position and little overlap in niche width between the years sampled. This likely contributed to making *P*. *electra’s* overall population isotopic niche width greater than that of *T*. *truncatus*, which showed no significant interannual variation in isotopic position. In 2010, the mean δ^15^N value of *P*. *electra* was significantly greater than in 2008 and 2011; however, its δ^13^C values did not vary significantly between years. This demonstrates the importance of timescale in measuring trophic niche [58]; if *P*. *electra* was only sampled in one year, it would appear to be much more of a specialist population with little intraspecific variation. The reason for its interannual differences, however, is not clear. It is possible that *P*. *electra* fed from relatively higher trophic position prey in 2010 (Fig 4), due either to differences in foraging behavior or differences in prey (e.g., with interannual shifts in the location of the North Equatorial current and countercurrent) or it could be an artifact of differential timing of sampling among years (e.g. changing position of ITCZ and isoscape). This result could alternatively be an artifact of them being absent in 2009; tissue samples may reflect them foraging in different areas altogether, given the several month lag time in assimilation likely for skin tissue. However, the consistency in δ^13^C values across years suggests that *P*. *electra’s* foraging habitat characteristics (distance from coast and depth) did not change significantly. *S*. *longirostris* was not analyzed for interannual variability as there were not enough samples to be confident in observed patterns.

We note that the single individual of *Orcinus orca* sampled from this system, who was part of a small group actively foraging on *T*. *truncatus* at the time of sampling, had a much higher δ^15^N value than any other delphinids. While any inferences must be limited given that this point is limited to a single sample, it is consistent with the ecological knowledge about *O*. *orca*. It is generally considered to be a top predator, but populations in temperate and polar ecosystems in both hemispheres are known to specialize on prey types: fish, pinnipeds, cetaceans [60]. Much less is known about the species in tropical latitudes but it is believed to be more of a generalist, though still a top predator [61].

## Conclusions

The results from isotopic analysis conducted in this study suggests that only subtle trophic partitioning occurs between the most dominant delphinid species at Palmyra Atoll. Most notably, there is some isotopic support for the claim that *T*. *truncatus* feeds at a higher trophic position than the other two species, which is possibly due to the different more diurnal feeding ecology, but this difference is very small. Interestingly, none of the species differed in their carbon isotopes, despite suggested differences in the amount of onshore vs. offshore and benthic vs. pelagic prey across species. There was also relatively little evidence for intraspecific variation in foraging ecologies across years among species. Only *P*. *electra* showed any differences at all in foraging ecologies, and these effects were relatively small and may simply be a sampling artifact due to differences in isoscapes across seasons or years.

The relatively low levels of isotopic differentiation shown among these predators was somewhat surprising as it seemed likely that such trophic partitioning of resources might facilitate the coexistence of these species, particularly among *P*. *electra* and *S*. *longirsotris* which share a general foraging strategy. It is possible that resources are not limiting in this system or at this life stage. While the highly oligotrophic nature of resources in this environment may make that seem unlikely, surveys of mesopelagic biomass in the area have shown high levels of offshore thermocline-associated communities [62]. Certainly, other marine predators in this system are known to finely partition trophic resources [9, 11]. It may be that there are other forms of behavioral partitioning of resources that have not yet been detected, or that there are other subtle aspects of food partitioning—e.g., among different prey species that school together—that are not detectable through relatively coarse isotopic approaches. However, isotopic niches are not always identical to trophic niches. Isotopic approaches may not be sufficiently resolved to detect subtle variations in trophic niche among species, either because different prey species were not sufficiently resolved in isotopic values, or because prey species were so varied in isotopic values as to make it difficult to detect a clear signal. Further research using approaches such as stable isotope mixing models and stomach content analysis will be critical to understand the means of coexistence among these regionally co-occurring species.

## Supporting information

S1 Fig Track lines by survey for Palmyra atoll. (JPG)

S1 Table Sample size by species, site, and year. (XLSX)

S1 File Data used in this study. (XLSX)
