# Supplementary figures and images for: Limited trophic partitioning among sympatric delphinids off a tropical oceanic atoll
Source: PLoS One. 2017 Aug 2;12(8):e0181526. doi: 10.1371/journal.pone.0181526 (PMC5540553; doi:10.1371/journal.pone.0181526)

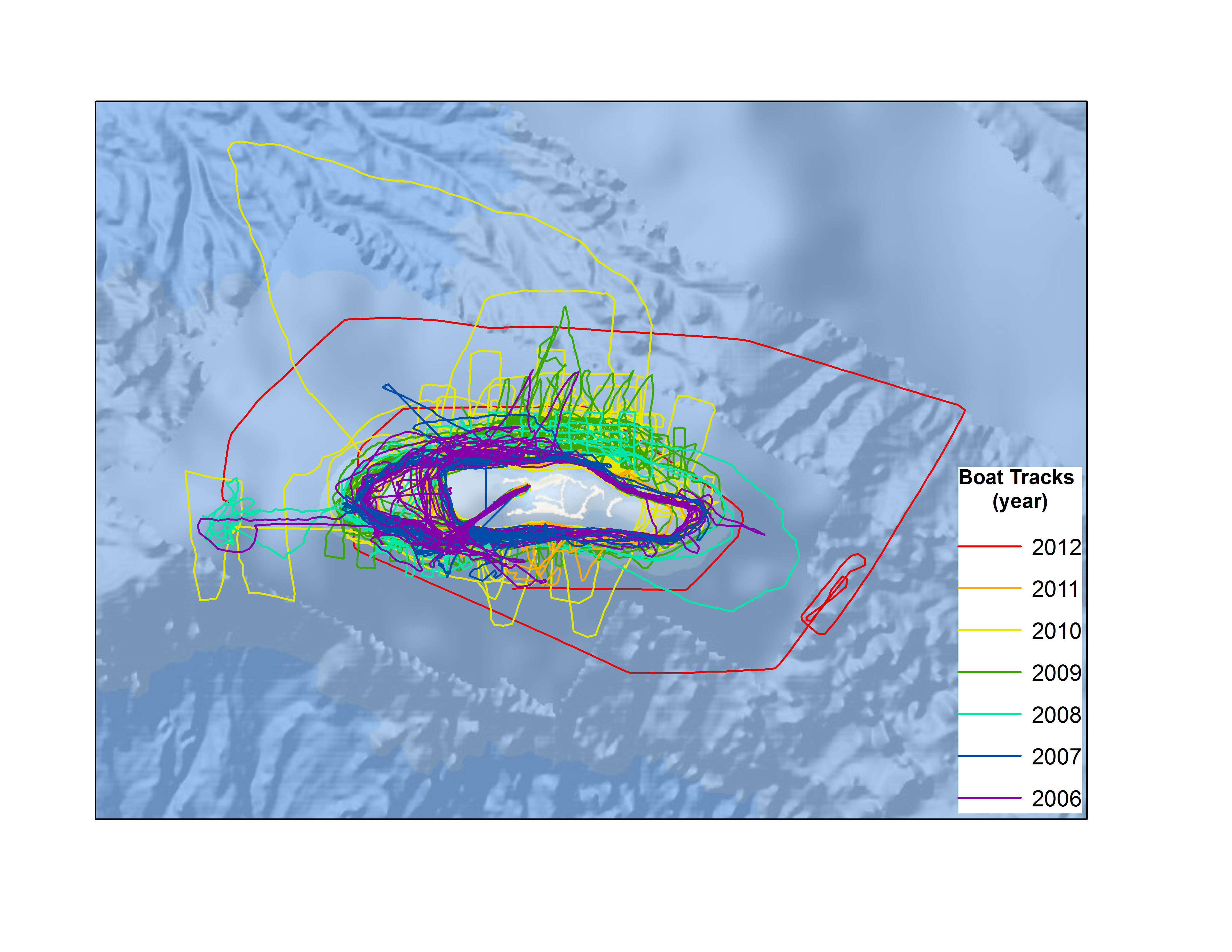

Supplement: S1 Fig — (JPG) [file pone.0181526.s001.jpg]
